# Mitochondrial genome of the *Harpiocephalus harpia* (Chiroptera: Vespertilionidae) from China

**DOI:** 10.1080/23802359.2020.1720539

**Published:** 2020-02-03

**Authors:** Xuan Tang, Wenhua Yu, Yi Wu

**Affiliations:** Key Laboratory of Conservation and Application in Biodiversity of South China, School of Life Sciences, Guangzhou University, Guangzhou, P. R. China

**Keywords:** Complete mitochondrial genome, *Harpiocephalus harpia*, Chiroptera, Vespertilio-nidae

## Abstract

In this study, a complete mitochondrial genome of a female *Harpiocephalus harpia* from Zhejiang Province, China, was sequenced using Illumina Hiseq. The genome is ∼16,400 bp in length, containing 13 protein-coding genes, 2 ribosomal RNA genes, 22 transfer RNA genes, and a control region. Most of the genes were encoded on the H-strand, except for 8 tRNA and *ND6* genes. Phylogenetic tree using maximum likelihood suggests *H. harpia* is sister taxon to *Murina.* The complete mitochondrial genome herein provides basic information for future taxonomic and phylogenetic studies.

Among all forest tube-nosed bats (Murininae), *Harpiocephalus harpia* (Chiroptera: Vespertilionidae) is the largest-sized and a phylogenetically basic species (Eger and Lim 2011; Simmons 2005; Soisook et al. 2013). Due to its strong sexual dimorphism, a long-term misclassification of *H. harpia* and *H. mordax* lasted until a comprehensive comparative analyses by Matveev (2005), which evidenced *H. mordax* is synonym to *H. harpia* (Corbet and Hill 1992; Matveev 2005; Simmons 2005). Nowadays, the species is widely distributed in Southeast Asia, including India, Myanmar, Vietnam, Laos, Indonesia, Malaysia, and the Philippines (Smith and Xie 2008; Wilson et al. 2019). In China, it is recorded in Yunnan (Wang 2003), Guangdong (Zhou et al. 2014), Fujian (Jiang et al. 2015), Taiwan (Lin et al. 2006), Guangxi (Chen et al. 2015), Jiangxi (Chen et al. 2015), Hainan ( Hu et al. 2018), Hunan (Yu et al. 2017), Guizhou (Gong et al. 2018), Zhejiang (Yue, Hu et al. 2019), Hubei (Yue, Hu et al. 2019). It is believed that it suffers from habitat destruction due to agricultural expansion and deforestation, thus IUCN Red List and China Species Red List (Wang and Xie 2009; Wilson et al. 2019) list it as Least Concern and Vulnerable, respectively.

In this study, we determined characteristics of the complete mitochondrial genome (Genbank accession No. MN885881) basing upon an adult female *H. harpia* captured from Dongyang City, Zhejiang Province, China (29°10′ 37″ N 120°30′ 43″ E ). Presently, the specimen is stored in the Key Laboratory of Conservation and Application in Biodiversity of South China, Guangzhou University, Guangdong, China (Accession ID GZHU 17323). Complete genomic DNA was extracted from 20 mg liver tissue using MiniBEST Universal Genomic DNA Extraction Kit (TAKARA, Dalian) and was sequenced using Illumina Hiseq sequencing technology. Illumina PE library (460 bp library) was constructed and the whole genome of mitochondrial was scanned by bioinformatics analysis after quality control of the obtained sequencing data.

Published mitochondrial genome of *M. ussuriensis* (Yoon et al. 2013), *M. leucogaster* (Yoon and Park 2016), *M. huttoni* (Zhang et al. 2016), *M. shuipuensis* (Huang et al. 2019) and *M. cyclotis* (Yue, Huang et al. 2019) were used to annotate the mitochondrial genome of *H. harpia*. The complete mitochondrial genome is ∼16,400 bp in length, containing 13 protein-coding genes, 22 tRNA genes, 2 rRNA genes, and 1 control region. Among these 37 genes, most genes were encoded on the H-strand while *ND6* (protein-coding gene) and eight tRNA genes (tRNA-Gln, Ala, Asn, Cys, Tyr, Ser, Glu, and Pro) were encoded on the L-strand. Among the 13 protein-coding genes, *ATP8* and *ATP6* were overlapped by 43 nucleotides, *ND4L* and *ND4* were overlapped by 7 nucleotides. The initiation codon of most of the protein-coding genes was ATG, except for those of *ND2* and *ND5* (ATA), *ND3* (ATC). Termination codon of *Cyt b* was AGA, seven protein-coding genes were TAA, while the rest were incomplete TA- (*ND1* and *ND3*) or T-- (*ND2*, *COX3*, *ND4*). Incomplete termination codon will probably be completed by polyA of the 3′ end of mRNA after transcription (Ojala et al. 1981). The length of the 22 tRNA genes range from 62 to 75 bp and can be folded into typical cloverleaf secondary structure, with an exception of tRNA-Ser. The 12S rRNA gene was located between tRNA-Phe and tRNA-Val and 16S rRNA gene was located between tRNA-Val and tRNA-Leu. Lengths of them were 971 and 1560 bp, respectively. The D-loop region is located between tRNA-Pro and tRNA-Phe.

For phylogenetic inference, 64 released complete mitochondrial genome sequences of Chiropteran species were downloaded from the GenBank. Sequence matrix was aligned using the MUSCLE (Edgar 2004). PartitionFinder 1.1.1 (Lanfear et al. 2012) was used to select the best partitioning scheme and best-fit models of nucleotide evolution. We inferred maximum-likelihood (ML) tree using RAxML 8.2.4 (Stamatakis 2006, 2014) with 100 bootstraps setting. Our ML tree shows that *H. harpia* and *Murina* clade is a sister group and support a monophyly of Murininae (Figure 1). The complete mitochondrial genome sequence of *H. harpia* herein could benefit the future taxonomic and phylogenetic studies.

**Figure 1.**
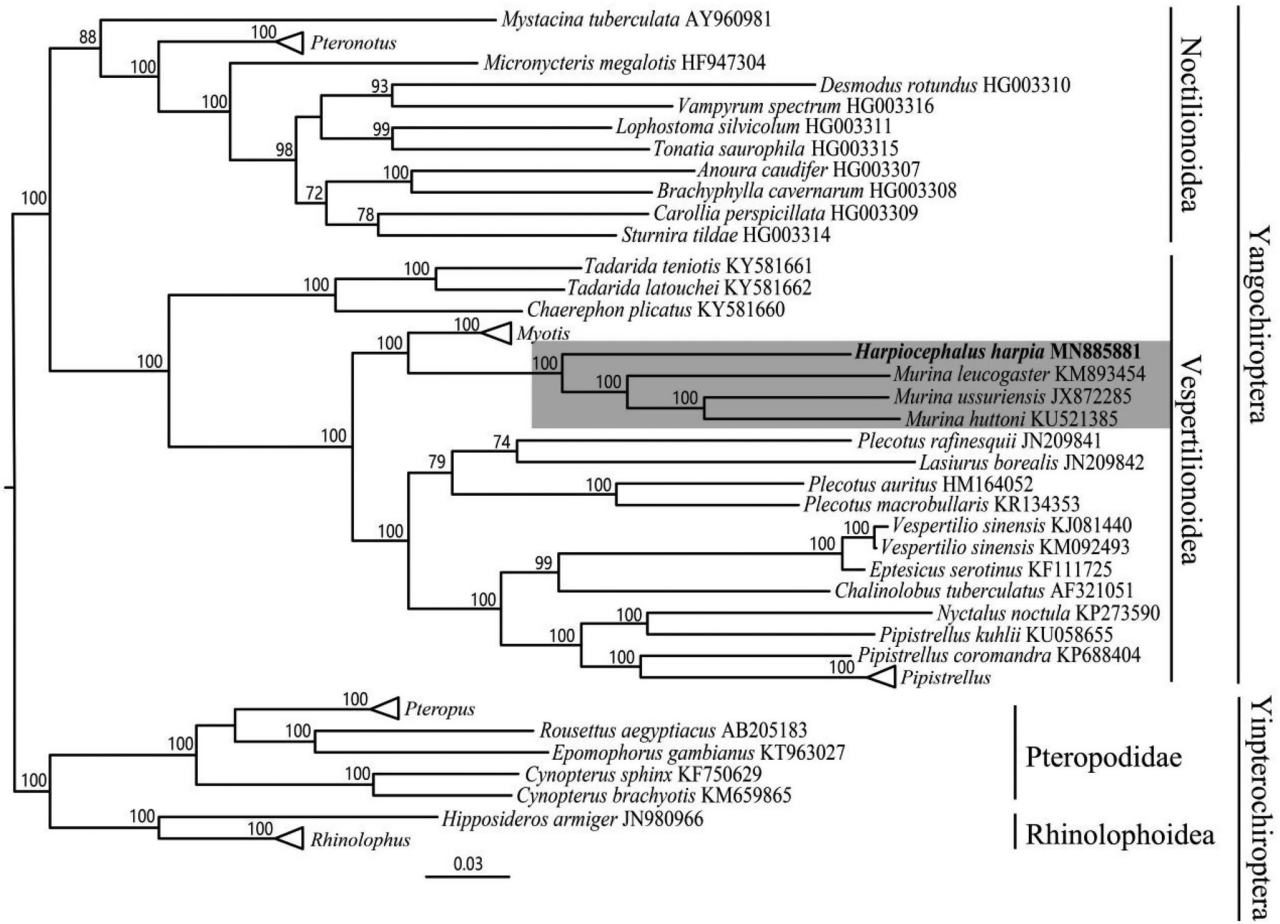
ML phylogenetic trees of 65 chiropteran species based on complete mitochondrial genome. Numbers above the nodes indicate boot strap values. Branch length is based on ML trees. The shaded highlights are our sample and *Murina*. Hollow triangles represent clusters of multiple species of *Pteropus*, *Rhinolophus*, *Pteronotus*, *Myotis* and *Pipistrellus* genus including 8, 7, 7, 7 and 3 species respectively.
